# P-456. Reduction in the incidence of chronic hepatitis C, liver cancer, and related healthcare burden following the introduction of interferon-free direct-acting antiviral therapy in Japan: a national claims database study

**DOI:** 10.1093/ofid/ofae631.656

**Published:** 2025-01-29

**Authors:** Naoki Kanda, Sinnpei Yamashita, Hideki Hashimoto, Hiromasa Yoshimoto, Kazuo Goda, Naohiro Mitsutakke, Shuji Hatakeyama

**Affiliations:** Division of General Medicine, Jichi Medical University, Shimotsuke, Tochigi, Japan; Division of General Medicine, Jichi Medical University, Shimotsuke, Tochigi, Japan; Division of General Medicine, Jichi Medical University, Shimotsuke, Tochigi, Japan; Institute for Health Economics and Policy, Minato-ku, Tokyo, Japan; Institute of Industrial Science, The University of Tokyo, Meguro-ku, Tokyo, Japan; Institute for Health Economics and Policy, Minato-ku, Tokyo, Japan; Division of General Medicine, Jichi Medical University, Shimotsuke, Tochigi, Japan

## Abstract

**Background:**

The World Health Organization aims to eliminate hepatitis C virus (HCV) infections by 2030. Interferon-free direct-acting antiviral (DAA) therapy, available in Japan since 2014, has demonstrated high hepatitis C cure rates and reduced hepatitis C-related hepatocellular carcinoma (HCC). However, DAA therapeutic efficacy on a national scale remains underexplored. In this study, we investigated national trends in HCV prevalence and complications, including liver cancer, during the era of interferon-free DAAs, with particular focus on the impact of coexisting human immunodeficiency virus (HIV) infection.

**Methods:**

We used the National Database of Health Insurance Claims, covering >98% of the population in Japan, to extract claims data on patients with chronic hepatitis C between 2013 and 2022. We identified patients with chronic hepatitis C annually based on the presence of corresponding diagnostic codes and relevant tests (abdominal ultrasound or HCC tumor markers) within each fiscal year. Next, we analyzed the trends in the prevalence of chronic hepatitis C (stratified by HIV status), DAA therapy, HCC incidence and treatment, and healthcare costs.

**Results:**

Between 2014 and 2022, 333,020 patients underwent interferon-free DAA therapy in Japan. The number of patients with chronic hepatitis C decreased from 690,336 in 2013 to 414,708 in 2022, representing a 40% prevalence reduction (95% confidence interval: 40%–40%). However, among people living with HIV (PLWH), we observed a 16% decline (10%–20%). Newly diagnosed HCC incidence among patients with chronic hepatitis C declined by 69% (68%–70%) over the 10 years. HCC treatment numbers also declined, with 50%, 80%, and 82% reductions in hepatectomies, radiofrequency ablation, and transarterial chemoembolization therapy, respectively. The total annual healthcare cost of HCV-infected individuals peaked in 2015 (1.1 trillion yen), coinciding with the widespread use of DAAs, and decreased to 65% of the 2013 expenses by 2022.

**Conclusion:**

The introduction of DAAs significantly reduced the HCV infection-related healthcare burden in Japan. Extensive screening and treatment, particularly in PLWH, are crucial for successful HCV eradication.

**Disclosures:**

**All Authors**: No reported disclosures

